# Supplementary material for: Sleep duration has a limited impact on the prevalence of menstrual irregularities in athletes: a cross-sectional study
Source: PeerJ. 2024 Feb 16;12:e16976. doi: 10.7717/peerj.16976 (PMC10875987; doi:10.7717/peerj.16976)
Supplement: Supplemental Information 2 [file peerj-12-16976-s002.docx]

library(tidyverse)

dat <- read.csv("data.csv")

dat_with_sleepdummy <-

dat %>%

mutate(SLEEP = case_when(sleep >= 6, "Normal",

sleep < 6, "Short"))
