# Supplementary material for: Sleep duration has a limited impact on the prevalence of menstrual irregularities in athletes: a cross-sectional study
Source: PeerJ. 2024 Feb 16;12:e16976. doi: 10.7717/peerj.16976 (PMC10875987; doi:10.7717/peerj.16976)
Supplement: Supplemental Information 4 [file peerj-12-16976-s004.doc]

STROBE Statement—Checklist of items that should be included in reports of ***cross-sectional studies***

|  | Item No | Recommendation | Relevant text from manuscript |
| --- | --- | --- | --- |
| **Title and abstract** | 1 | (*a*) Indicate the study’s design with a commonly used term in the title or the abstract | Sleep duration has a limited impact on menstrual irregularities in athletes: a cross-sectional study (TITLE) |
|  |  | (*b*) Provide in the abstract an informative and balanced summary of what was done and what was found | This study aimed to investigate the relationship between menstrual irregularities, energy intake, and sleep deprivation among female athletes. The athletes are divided into two tires: Highly Trained/National Level (Tier 3) and Elite/International Level (Tier 4).  A total of 128 female athletes, with an average age of 19.2 ± 1.2 years, participated in the study and tracked their food intake over a three-day period. Menstrual status and sleep duration were assessed using a questionnaire, and psychological anxiety was evaluated using the State and Trait Anxiety Inventory (STAI). (Abstract>Background)  These findings emphasize the crucial role of maintaining favorable mental health conditions, characterized by reduced state anxiety, in preserving normal menstruation among female athletes aged 15 - 23 years. This study provides new insights, suggesting that high state anxiety may exert a greater impact on menstrual irregularities in female athletes compared to sleep duration. (Abstract>Conclusions) |
| Introduction | | |  |
| Background/rationale | 2 | Explain the scientific background and rationale for the investigation being reported | Previous research has shown that insufficient nutritional intake, particularly a deficiency in carbohydrates and vitamin D, also play a significant role in the occurrence of menstrual irregularities among athletes (Miyamoto et al., 2021a, 2022). (L5-8)  The recent evidence suggests that sleep disturbances, including shortened sleep duration and insomnia, may contribute to menstrual cycle alterations and the development of menstrual disorders (Kim et al., 2018). A study conducted in the general female population reported that individuals who sleep 5 hours or less have a higher risk of experiencing irregular menstruation compared to those who sleep 6 hours or more (Kim et al., 2018). (L.21-25)  Psychological anxiety, sleep deprivation, and energy intake may play a combined role in menstrual irregularities. Therefore, this study aims to assess the relative impact of mental anxiety, sleep deprivation, and energy intake on menstrual function in top-level female athletes. (L31-34) |
| Objectives | 3 | State specific objectives, including any prespecified hypotheses | We hypothesize that sleep deprivation may not directly affect menstrual irregularities but may contribute to psychological anxiety and inadequate energy intake, resulting in menstrual irregularities in elite-level female athletes. In other words, we expect that heightened psychological anxiety will be associated with an elevated risk of menstrual irregularities, and that shortened sleep duration will also contribute to alterations in the menstrual irregularities. Additionally, previous studies have provided mixed findings regarding the relationship between short sleep duration and energy intake, with some reports suggesting a connection and others finding no significant association. In summary, this study aimed to clarify the relative impact of psychological anxiety, sleep deprivation, and energy intake on menstrual irregularities in elite-level female athletes. (L35-44) |
| Methods | | |  |
| Study design | 4 | Present key elements of study design early in the paper | This study was structed using a cross-sectional design and conducted over 6 months. (L48) |
| Setting | 5 | Describe the setting, locations, and relevant dates, including periods of recruitment, exposure, follow-up, and data collection |  |
| Participants | 6 | (*a*) Give the eligibility criteria, and the sources and methods of selection of participants | The present included 140 female athletes, aged 15 and 23 years, competing at the inter-college level in track and field events and at the international level in rowing, corresponding to “Tier 3” and “Tier 4” categories as defined by Mckay et al. (2022). The study included athletes from the Japanese national rowing team and long-distance runners from a university track and field club, all of whom were at least one-year post-menarche. All athletes were naturally menstruating and were not using non-hormonal contraceptives. All athletes affiliated with those teams expressed their willingness to participate in these surveys and took parts. (L48-57) |
| Variables | 7 | Clearly define all outcomes, exposures, predictors, potential confounders, and effect modifiers. Give diagnostic criteria, if applicable | Participants were categorized into two groups based on their sleep duration: those sleeping less than 6 hours per day (the Short group, defined as experiencing sleep deprivation^18^) and those sleeping more than 6 hours per day (the Normal group), following criteria used in a previous study (Krishnan et al., 2016). The impact of sleep status on state anxiety and daily energy intake was examined using Analysis of Variance (ANOVA).  12 participants with incomplete data were subsequently excluded from the analysis. (L105-109) |
| Data sources/ measurement | 8* | For each variable of interest, give sources of data and details of methods of assessment (measurement). Describe comparability of assessment methods if there is more than one group | The present included 140 female athletes, aged 15 and 23 years, competing at the inter-college level in track and field events and at the international level in rowing, corresponding to “Tier 3” and “Tier 4” categories as defined by Mckay et al. (2022). (L105-108) |
| Bias | 9 | Describe any efforts to address potential sources of bias | All athletes affiliated with those teams expressed their willingness to participate in these surveys and took parts. The mean age of the participants was 19.2 ± 1.2 years. Prior to their involvement, all athletes provided informed consent. (L56-58) |
| Study size | 10 | Explain how the study size was arrived at | The appropriate sample size (n = 107) was calculated using the following: odds ratio = 2, H0 = 0.15, α = 0.05, power = 0.8, and X distribution = normal on logistic regression. (L51-53) |
| Quantitative variables | 11 | Explain how quantitative variables were handled in the analyses. If applicable, describe which groupings were chosen and why | A generalized linear model (GLM) with a log link function was employed to investigate the effects of sleep deprivation on the presence of menstrual irregularities. The sleep deprivation data comprised of binary values (0 and 1) representing “normal” and “sleep deprivation”. The menstrual irregularity data comprised of binary values (0 and 1) representing “normal menstruation” and “menstrual irregularity” states. GLMs were also used to assess the effects of state anxiety, sleep status, and energy intake (total daily caloric intake) on menstrual irregularities. The selection of independent variables in this analysis, including state anxiety, sleep status, energy intake, and carbohydrate intake, was guided by previous research (Schlip et al., 2015; Kim et al., 2018), and determined using the Akaike information criterion (AIC). A significance level of 5% was adopted for all analyses.  (L109-119) |
| Statistical methods | 12 | (*a*) Describe all statistical methods, including those used to control for confounding | A generalized linear model (GLM) with a log link function was employed to investigate the effects of sleep deprivation on the presence of menstrual irregularities. The sleep deprivation data comprised of binary values (0 and 1) representing “normal” and “sleep deprivation”. The menstrual irregularity data comprised of binary values (0 and 1) representing “normal menstruation” and “menstrual irregularity” states. GLMs were also used to assess the effects of state anxiety, sleep status, and energy intake (total daily caloric intake) on menstrual irregularities. The selection of independent variables in this analysis, including state anxiety, sleep status, energy intake, and carbohydrate intake, was guided by previous research (Schlip et al., 2015; Kim et al., 2018), and determined using the Akaike information criterion (AIC). A significance level of 5% was adopted for all analyses.  (L109-111) |
|  |  | (*b*) Describe any methods used to examine subgroups and interactions |  |
|  |  | (*c*) Explain how missing data were addressed |  |
|  |  | (*d*) If applicable, describe analytical methods taking account of sampling strategy |  |
|  |  | (*e*) Describe any sensitivity analyses |  |
| Results | | |  |
| Participants | 13* | (a) Report numbers of individuals at each stage of study—eg numbers potentially eligible, examined for eligibility, confirmed eligible, included in the study, completing follow-up, and analysed | Table 1 summarizes the physical characteristics of the athletes (n = 128). (128) |
|  |  | (b) Give reasons for non-participation at each stage |  |
|  |  | (c) Consider use of a flow diagram |  |
| Descriptive data | 14* | (a) Give characteristics of study participants (eg demographic, clinical, social) and information on exposures and potential confounders | The present included 140 female athletes, aged 15 and 23 years, competing at the inter-college level in track and field events and at the international level in rowing, corresponding to “Tier 3” and “Tier 4” categories as defined by Mckay et al. (2022). However, 12 participants with incomplete data were subsequently excluded from the analysis. (L49-51; L62-63) |
|  |  | (b) Indicate number of participants with missing data for each variable of interest |  |
| Outcome data | 15* | Report numbers of outcome events or summary measures |  |
| Main results | 16 | (*a*) Give unadjusted estimates and, if applicable, confounder-adjusted estimates and their precision (eg, 95% confidence interval). Make clear which confounders were adjusted for and why they were included | The GLM analysis revealed a significant association between an increase in state anxiety and the presence of menstrual irregularity (z = 2.000, p = 0.048, see Table4). Additionally, a decrease in sleep duration did not significantly impact the prevalence of menstrual irregularities (z = 1.493, p = 0.136, see Table 4). Subsequently, we observed that dietary energy intake did not have a significant impact the presence of menstrual irregularities (z = 1.029, p = 0.303, see Table 4).  (L139-144) |
|  |  | (*b*) Report category boundaries when continuous variables were categorized |  |
|  |  | (*c*) If relevant, consider translating estimates of relative risk into absolute risk for a meaningful time period |  |
| Other analyses | 17 | Report other analyses done—eg analyses of subgroups and interactions, and sensitivity analyses |  |
| Discussion | | |  |
| Key results | 18 | Summarise key results with reference to study objectives | The present study reveals that among female athletes aged 15-23 years, no significant trend linking short sleep duration and energy intake to menstrual irregularities was observed. However, heightened psychological anxiety had a stronger influence on the presence of menstrual irregularities compared to insufficient sleep duration and energy intake. (L149-150) |
| Limitations | 19 | Discuss limitations of the study, taking into account sources of potential bias or imprecision. Discuss both direction and magnitude of any potential bias | There are several potential limitations that should be acknowledged in this study. Firstly, data on menstrual cycles and sleep duration were obtained through a self-administered questionnaire, potentially introducing recall bias. The actual sleep duration is unknown as the survey relied on a questionnaire. Furthermore, this study did not account for the time of naps. In the future, we believe that verification using a device that can accurately determine sleep time is needed. Additionally, the definition of menstrual irregularity used in this study differed from the definition from the definition of intervals of 35 days or longer as previously defined in previous studies (McNulty et al., 2020). As noted in the Methods section, this difference in definition should not affect the results of this study because all participants with intervals of 35 days or more had menstrual cycle of 2 months or longer. While the STAI was employed to assess state and trait anxiety in this study, future investigations may consider incorporating alternative measures of mental stress. The STAI, widely used clinically and featuring a well-defined clinical cutoff line (> 40), was utilized to assess psychological anxiety (Grant et al., 2008) revealing that athletes in our study had high levels of psychological anxiety. In fact, 78 of 128 athletes had state anxiety over 40, and 94 of 128 athletes had trait anxiety over 40. It can be inferred that the athletes had higher scores compared to the general population, although a direct comparison was not made in this study. The findings of the present study also indicate that female athletes may experience menstrual irregularities in the presence of heightened psychological anxiety. Further validation with large-scale data is warranted to gain a deeper understanding of these mechanisms and their implications.  (L187-206) |
| Interpretation | 20 | Give a cautious overall interpretation of results considering objectives, limitations, multiplicity of analyses, results from similar studies, and other relevant evidence | This study provides novel insights suggesting that high state anxiety may have a stronger influence on menstrual irregularities in female athletes compared to sleep duration. (L.207-208) |
| Generalisability | 21 | Discuss the generalisability (external validity) of the study results | The influence of energy intake remains uncertain in this study due to the lack of precise determination regarding energy expended during exercise. Although this study’s findings do not definitively rule out the possibility that sleep duration contributed to psychological anxiety, which subsequently led to menstrual irregularities, further studies will provide deeper insight into this matter. (L182-186) |
| Other information | | |  |
| Funding | 22 | Give the source of funding and the role of the funders for the present study and, if applicable, for the original study on which the present article is based | There is no funding support in the present study. (L211) |

*Give information separately for exposed and unexposed groups.

**Note:** An Explanation and Elaboration article discusses each checklist item and gives methodological background and published examples of transparent reporting. The STROBE checklist is best used in conjunction with this article (freely available on the Web sites of PLoS Medicine at http://www.plosmedicine.org/, Annals of Internal Medicine at http://www.annals.org/, and Epidemiology at http://www.epidem.com/). Information on the STROBE Initiative is available at www.strobe-statement.org.
